# Development of cannabis use disorder in medical cannabis users: A 9-month follow-up of a randomized clinical trial testing effects of medical cannabis card ownership

**DOI:** 10.3389/fpsyt.2023.1083334

**Published:** 2023-03-07

**Authors:** Megan E. Cooke, Kevin W. Potter, Julia Jashinski, Michael Pascale, Randi M. Schuster, Brenden Tervo-Clemmens, Bettina B. Hoeppner, Gladys N. Pachas, A. Eden Evins, Jodi M. Gilman

**Affiliations:** ^1^Department of Psychiatry, Center for Addiction Medicine (CAM), Massachusetts General Hospital (MGH), Boston, MA, United States; ^2^Department of Psychiatry, Harvard Medical School, Boston, MA, United States; ^3^Athinoula A. Martinos Center for Biomedical Imaging, Department of Radiology, Massachusetts General Hospital (MGH), Harvard Medical School, Charlestown, MA, United States

**Keywords:** cannabis (marijuana), insomnia, pain, anxiety, depression, cannabis use disorder, medical cannabis, medical marijuana

## Abstract

**Background:**

Evidence for long-term effectiveness of commercial cannabis products used to treat medical symptoms is inconsistent, despite increasingly widespread use.

**Objective:**

To prospectively evaluate the effects of using cannabis on self-reported symptoms of pain, insomnia, anxiety, depression, and cannabis use disorder (CUD) after 12 months of use.

**Methods:**

This observational cohort study describes outcomes over 9 months following a 12-week randomized, waitlist-controlled trial (RCT: NCT*03224468*) in which adults (*N* = 163) who wished to use cannabis to alleviate insomnia, pain, depression, or anxiety symptoms were randomly assigned to obtain a medical marijuana card immediately (immediate card acquisition group) or to delay obtaining a card for 12 weeks delay (delayed card acquisition group). During the 9-month post-randomization period, all participants could use cannabis as they wished and choose their cannabis products, doses, and frequency of use. Insomnia, pain, depression, anxiety, and CUD symptoms were assessed over the 9-month post-randomization period.

**Results:**

After 12 months of using cannabis for medical symptoms, 11.7% of all participants (*n* = 19), and 17.1% of those using cannabis daily or near-daily (*n* = 6) developed CUD. Frequency of cannabis use was positively correlated with pain severity and number of CUD symptoms, but not significantly associated with severity of self-reported insomnia, depression, or anxiety symptoms. Depression scores improved throughout the 9 months in all participants, regardless of cannabis use frequency.

**Conclusions:**

Frequency of cannabis use was not associated with improved pain, anxiety, or depression symptoms but was associated with new-onset cannabis use disorder in a significant minority of participants. Daily or near-daily cannabis use appears to have little benefit for these symptoms after 12 months of use.

## 1. Introduction

With growing state-level legalization of commercial cannabis markets, individuals are increasingly using cannabis products hoping to alleviate symptoms of various chronic medical conditions (1–3). In most US states, people seeking cannabis for medical or psychiatric symptoms must obtain state-specific medical cannabis authorization cards to purchase cannabis products from dispensaries (4, 5). Enrollment in medical cannabis programs increased 4.5-fold from 2016 to 2020 (6). While interest in using commercial cannabis products for medical conditions is high, rigorous data on its safety and effectiveness for symptom relief is sparse (7), and few studies assess longer-term outcomes.

The most common conditions for which individuals obtain medical cannabis cards are pain, insomnia, anxiety and depressed mood (8–10), but evidence for the efficacy of cannabis to treat these symptoms has been mixed (7, 11–18). Studies examining the effects of cannabis on chronic pain have generally had small sample sizes and null results (19), except for some evidence of efficacy for neuropathic pain (19–21). While patients using opioid medications for chronic pain have reported preliminary success in substituting cannabis for these medications (22, 23), electronic health records, including prescription drug monitoring program data from a large multisite medical cannabis program, demonstrated minimal to no change in either opioids or sedative hypnotics over the 6 months of medical cannabis use (24). An ongoing randomized controlled trial is currently assessing the effectiveness of cannabis for pain control and opioid dose reduction (25). A relationship between cannabis use and sleep has been theorized based on connections between the endocannabinoid system and circadian rhythms (26, 27) with research indicating improved sleep in the short term (28–31), but a disruption in sleep quality over long term use (32, 33). The use of cannabis as a therapeutic for anxiety or depression is controversial, as existing trials are limited by small sample sizes, as well as deficits in the overall study designs, which limit the clinical applications of findings (34, 35).

Unlike Food and Drug Association (FDA) approved medications, treatments approved by voter initiatives or legislative action come with little evidence to guide dosing to optimize benefits and minimize adverse effects. Further, recent US national data reports that 3 in 10 adults who use cannabis develop cannabis use disorder (CUD), with 23% developing severe CUD (36) often with tolerance to Δ9-tetrahydrocannabinol (THC) and withdrawal symptoms (37, 38). Data are lacking on whether adults using cannabis for medical purposes develop similar rates of CUD to those who use cannabis for recreational purposes. Rigorous studies of the effects of cannabis use on clinical outcomes will be critical to inform patient and clinician decision-making.

This study describes a prospective, 9-month follow-up of participants enrolled in a randomized clinical trial (RCT; *NCT03224468*) in which adults seeking cannabis to alleviate pain, insomnia, anxiety, or depression were randomized to immediate card acquisition or 12 weeks delayed card acquisition groups. In the 12-week RCT, immediate cannabis card acquisition was associated with developing CUD, improved self-reported insomnia, and no change in pain, depression, or anxiety symptoms (29). Here, we report cannabis use frequency, CUD, pain, insomnia, anxiety, and depression symptoms over the 9 months following the 12-week randomized phase. Based on results of the RCT, we hypothesized that after 12 months of cannabis use, symptoms of insomnia would improve, but symptoms of CUD would increase. We did not hypothesize any changes in pain, depression, or anxiety symptoms.

## 2. Methods

This study was approved by the Massachusetts General Brigham Institutional Review Board. All participants provided informed consent. Participants were financially compensated for their time, but the study did not provide or pay for the medical cannabis cards or any cannabis products. Adults without CUD seeking to obtain a medical cannabis card for pain, insomnia, anxiety, or depressive symptoms participated in a 12-week, single-blind randomized pragmatic clinical trial (*NCT03224468*), described previously (29), in which they were assigned to either obtain a card immediately or to delay card acquisition by 12 weeks. Participants assigned to the immediate card acquisition group were required to obtain a card to participate in the study. All participants were then followed for a 9-month period in which all could obtain medical cannabis cards if they desired, and use the cannabis products of their choice, dose, and frequency following the randomized phase.

### 2.1. Design

Participants completed assessments of clinical symptoms (pain, insomnia, anxiety, depression), cannabis use and CUD at baseline and weeks 2, 4, and 12 of the randomized phase. During the follow-up period, participants completed assessments of clinical symptoms at months 6 and 12 and of cannabis use monthly.

### 2.2. Participants

Participants were men and women aged 18–65 (inclusive) who expressed an interest in using cannabis to alleviate symptoms of pain, insomnia, anxiety, or depression and were recruited through community advertising; a full description of the sample is reported elsewhere (29). Exclusion criteria included daily or near-daily cannabis use in the prior 3 months, diagnosis of current CUD, other substance use disorder, or serious unstable medical condition at screening or baseline assessments.

### 2.3. Measurements and outcomes

Cannabis use frequency was collected monthly *via* REDCap using a 7-point ordinal rating scale. Due to low cell counts, we collapsed the scale down to four ratings: (a) 5–7 days per week, (b) 1–4 days per week, (c) less than once a week, and (d) less than once a month.

We report results for five clinical outcomes. Pain in the past 24 h was assessed by the Pain Severity subscale of the Brief Pain Inventory Short Form (BPI-PS) (39) on a 0–10 point scale (0 = no pain, 10 = worst pain imaginable). Insomnia in the past month was assessed by the Athens Insomnia Scale (AIS) (40) on a 0–24 point scale, with higher scores indicating more severe sleep difficulties. Anxiety and depressive symptoms in the past week were assessed using the corresponding subscales of the Hospital Anxiety and Depression Scale (HADS) (41), each on a 0–21 point scale, with scores of 8–10 indicating borderline abnormal and a score of 11 or greater indicating abnormal levels of anxiety or depression for a given subscale. Cannabis use disorder (CUD) symptoms were assessed in interviews by doctoral-level or registered nurse investigators blinded to group assignment using the CUD Checklist of the Diagnostic and Statistical Manual of Mental Disorders (42), with scores ranging from 0 to 11 (with 2 or more symptoms indicating a CUD diagnosis, and higher scores indicated more severe CUD).

### 2.4. Analytic plan

All analyses examined how clinical outcomes (cannabis use frequency, symptoms of pain, insomnia, anxiety, depression, and CUD) changed from the end of the RCT to the end of the follow-up period (month 12). Time was assessed *via* a linear trend, using the number of months since enrollment per participant (accounting for individual variation in the timing of study visits). All analyses included a participant-varying intercept and slope for the time trend. Analyses also included a covariate for a participant's symptom levels at baseline. We used a dummy-coded variable for randomization group (immediate = 1, delayed = 0).

We first assessed change in cannabis use over time, testing for differences by randomization group and for a group by time interaction. We fit the ordinal cannabis use outcome (the 4-point rating scale) using a multi-level cumulative probit regression (43). Analyzing the ordinal ratings using the cumulative probit model avoided systematic errors caused by analyzing ordinal ratings using linear regression (44).

We next assessed change in symptom levels for the five clinical outcomes (symptoms of pain, insomnia, anxiety, depression, and CUD) over time, again testing for differences by randomization group and for a group by time interaction. We fit the clinical inventory scores and CUD symptom counts using a multi-level beta-binomial regression model.

Finally, we reassessed change in symptom levels for the five clinical outcomes over time based on cannabis use frequency, regardless of randomization group. Here, we used an approach based on projective inference (45), fitting as a reference model a cumulative probit regression with subject-varying intercepts and slopes for change over time (expanded to capture linear, quadratic, and cubic trends) applied to participants' full set of 10 monthly cannabis use ratings. We used this reference model to interpolate continuous estimates of cannabis use at months 3, 6, and 12 (when clinical outcomes were collected).

All analyses were conducted in a Bayesian framework, allowing implementation of complex statistical models and intuitive interpretations of uncertainty intervals and p-values as the probability of a test statistic given the data and prior assumptions (46). To address the potential for an inflated risk of false positives (47), we used a model-averaging approach (48), in which results across nested models [i.e., models for (1) a main effect of time, (2) main effects of both time and group/cannabis use, or (3) their interaction] are averaged together based on their predictive utility [e.g., stacking weights based on leave-one-out cross-validation; (49)]. We report estimated standardized effect sizes (ES, mean differences scaled by baseline standard deviations), 95% credible intervals, and posterior p-values. All results are from the model-averaged adjusted estimates. Effects were deemed statistically significant if adjusted posterior *p* < 0.05.

## 3. Results

Among the 186 participants enrolled in the original clinical trial, 163 had at least one follow-up assessment (at either 6 or 12 months) with complete data for all five clinical outcomes and for cannabis use. The analytic sample was 68.1% female, 82.2% white, and had an average age of 37.3 (SD = 14.4) years. See Table 1 for additional descriptive characteristics measured at baseline. Participants in the immediate acquisition group were required to obtain a medical cannabis card to be eligible for the clinical trial, thus all (100%) obtained a card. In contrast, only 36.5% of participants assigned to delayed acquisition obtained a card by the 12-month timepoint. Although the majority of participants in the delayed acquisition group did not obtain a card, 74.6% reported using cannabis 1 or more days per week for at least a month during the follow-up period (months 3–12).

**Table 1:** Participant characteristics at baseline.

**Measure**	**Overall**	**Immediate**	**Delayed**	***p*-value**
Sample size	163	96	67	
Finished only 1 follow-up visit	11.7% (19)	9.4% (9)	14.9% (10)	*p* = 0.667
Age; M (SD)	37.3 (14.4)	38.4 (14.4)	35.7 (14.4)	*p* = 0.930
Biological sex at birth; % (*n*)				
Female	68.1% (111)	68.8% (66)	67.2% (45)	*p* = 0.904
Male	31.9% (52)	31.2% (30)	32.8% (22)	
Race; % (*n*)	
Asian	6.1% (10)	6.2% (6)	6% (4)	*p* = 0.787
Black or African American	6.7% (11)	6.2% (6)	7.5% (5)	*p* = 0.716
Multi-racial	2.5% (4)	3.1% (3)	1.5% (1)	*p* = 0.870
Not listed	2.5% (4)	1% (1)	4.5% (3)	
White	82.2% (134)	83.3% (80)	80.6% (54)	*p* = 0.910
Hispanic or Latino; % (*n*)	4.9% (8)	4.2% (4)	6% (4)	*p* = 0.624
Education level; % (*n*)				*p* = 0.600
High school	3.7% (6)	3.1% (3)	4.5% (3)	
Part college	19.6% (32)	15.6% (15)	25.4% (17)	*p* = 0.674
College 2–4 years	35.6% (58)	36.5% (35)	34.3% (23)	*p* = 0.911
Part grad school	40.5% (66)	44.8% (43)	34.3% (23)	*p* = 0.978
Unknown	0.6% (1)	0% (0)	1.5% (1)	
Education years; M (SD)	16.5 (2.3)	16.6 (2.3)	16.3 (2.3)	*p* = 0.989

**Figure 1:**
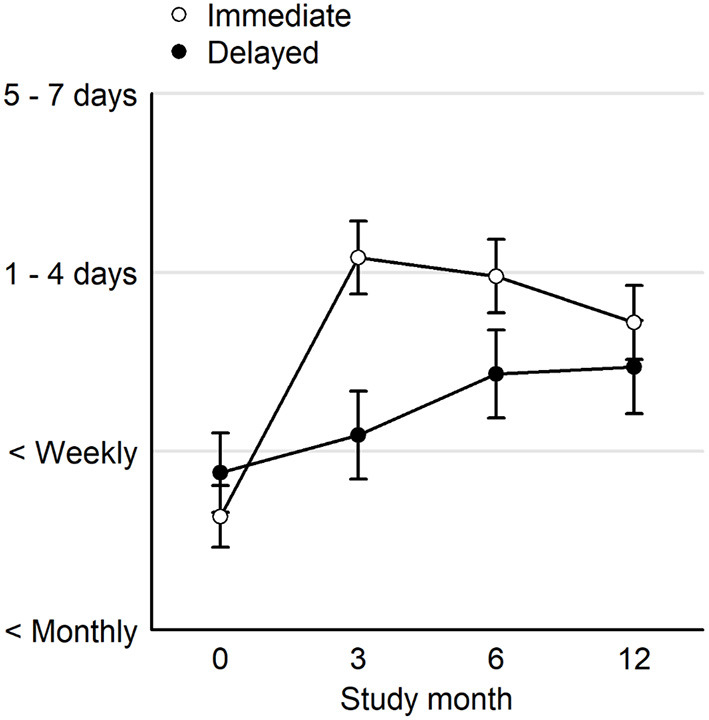
Averages and 95% uncertainty intervals over the 4-point ordinal rating for cannabis use at the start of the clinical trial (Month 0), the end of the clinical trial (Month 3), and the follow up period (Months 6 and 12). Immediate, immediate card acquisition group; Delayed, delayed card acquisition group.

As previously reported (29), at the end of the clinical trial (month 3) the immediate acquisition group had higher rates of cannabis use compared to the delayed acquisition group (β = 1.49, CI = 0.99–2.00, post. *p* < 0.001). However, by month 12 the immediate acquisition group had reduced cannabis use (β = −0.50, CI = −0.88 to −0.08, post. *p* = 0.020), while the delayed acquisition group had a slight increase in cannabis use frequency (β = 0.38, CI = −0.15–0.86, post. *p* = 0.132). The immediate acquisition group still had greater use than the delayed group at month 12 (β = 0.61, CI = 0.01–1.31, post. *p* = 0.046) (Figure 1, Supplementary Table 1).

At month 12, 11.7% (*n* = 19) of participants, and 17.1% of those using cannabis daily or near-daily (*n* = 6) met DSM-V diagnostic criteria for CUD, defined as 2 or more symptoms of CUD; most had mild (*n* = 15), defined as 2–3 symptoms, two participants had moderate, defined as 4–5 symptoms, and two participants had severe CUD, defined as 6 or more symptoms. For those with a CUD diagnosis, the most frequently reported CUD symptoms were tolerance (58%), using despite experiencing problems (44%), spending a lot of time using (31%), and craving (31%). The most common combinations of symptoms were tolerance combined with either craving (10%), using more than intended (8%), or wanting to cut back (6%) (Supplementary Figure 1). There was no statistically significant effect of group on number of CUD symptoms by the 12-month timepoint (ES = 0.63, 95% CI = 0.00–1.31, post. *p* = 0.185).

Averaging over time points, the immediate acquisition group had lower AIS scores (ES = 0.30, −0.53 to −0.08, *p* = 0.008) and higher BPI scores (ES = 0.15, 0.03–0.27, *p* = 0.012) compared to the delayed acquisition group. There were, however, no statistically significant time by group interactions on any outcome (Supplementary Table 2, Supplementary Figure 2), indicating that although there was a main effect of group, the groups did not differ in how their clinical symptoms changed over time. Depression scores improved from month 3 to month 12, regardless of randomization group or frequency of cannabis use (ES = −0.13, 95% CI = −0.26 to −0.01, post. *p* = 0.032). Pain, insomnia, and anxiety symptoms did not change significantly over the follow up period (Table 2).

**Table 2:** Symptoms of insomnia, pain, depression, anxiety, and CUD during the 9-month post-randomization period.

**Outcome**	**Study month**	**Mean (SD); *N***
HADS [Anxiety]	3	6.6 (4.1); 163
	6	6.8 (4.3); 163
	12	6.2 (4.2); 149
Main effect of time		*p* = 0.109
HADS [Depression]	3	4.3 (3.8); 163
	6	4.0 (3.6); 163
	12	3.8 (3.5); 149
**Main effect of time**		***p*** **=** **0.032**
AIS	3	7.9 (4.9); 163
	6	7.5 (4.8); 163
	12	7.3 (4.6); 149
Main effect of time		*p* = 0.182
BPI [Severity]	3	1.58 (2.18); 163
	6	1.30 (2.03); 163
	12	1.57 (2.16); 149
Main effect of time		*p* = 0.891
CUD symptoms	3	0.40 (0.83); 163
	6	0.48 (0.91); 163
12	0.48 (1.02); 149
Main effect of time		*p* = 0.695

HADS, Hospital Anxiety and Depression Scale; AIS, Athens Insomnia Scale; BPI, Brief Pain Inventory; CUD, Cannabis Use Disorder, bolded p-values indicate a statistically significant change in symptoms over the 9-month follow-up period.

More frequent cannabis use was associated with greater pain (ES = 0.07, 95% CI = 0.02–0.12, post. *p* = 0.006) and more CUD symptoms (ES = 0.55, 95% CI = 0.31–0.86, post. *p* < 0.001). More frequent cannabis use was not associated with improvement in insomnia, depression, or anxiety. Those who used cannabis 3 or more days per week were 2.69 times more likely to develop CUD, with disorder rates of 15.4% for those who used 3 or more days compared to disorder rates of 5.6% for those who used <3 days. We found no statistically significant time by cannabis use interactions across any outcome (Table 3).

**Table 3:** Main effects of time and cannabis use frequency and their interaction for each clinical outcome.

**Outcome**	**Effect**	**Cohen's D Mean; 95% CI**	**Post. *p*-value**
HADS [Anxiety]	Main effect of time	−0.11; −0.24–0.03	*p* = 0.111
	Main effect of cannabis use	−0.07; −0.18–0.00	*p* = 0.301
	Time x cannabis use interaction	0.08; 0.00–0.24	*p* = 0.338
HADS [Depression]	**Main effect of time**	–**0.14;** –**0.26**- –**0.01**	***p*** **=** **0.030**
	Main effect of cannabis use	0.00; 0.00–0.00	*p* = 0.990
	Time x cannabis use interaction	0.00; 0.00 to 0.00	p = 0.990
AIS	Main effect of time	−0.11; −0.26–0.04	*p* = 0.149
	Main effect of cannabis use	−0.08; −0.23–0.00	*p* = 0.454
	Time x cannabis use interaction	0.00; 0.00–0.02	*p* = 0.530
BPI [Severity]	Main effect of time	0.00; −0.08–0.08	*p* = 0.952
	**Main effect of cannabis use**	**0.07; 0.02**-**0.12**	***p*** **=** **0.006**
	Time x cannabis use interaction	0.00; −0.03–0.02	*p* = 0.951
CUD symptoms	Main effect of time	0.07; −0.35–0.50	*p* = 0.733
	**Main effect of cannabis use**	**0.55; 0.31**-**0.86**	***p*** **<** **0.001**
	Time x cannabis use interaction	0.04; −0.21–0.27	*p* = 0.736

HADS, Hospital Anxiety and Depression Scale; AIS, Athens Insomnia Scale; BPI, Brief Pain Inventory; CUD, Cannabis Use Disorder. Bolded p-values indicate a statistically significant effect.

## 4. Discussion

In this 9-month prospective follow-up analysis of a 12-week RCT of immediate or delayed medical cannabis card acquisition, greater cannabis use frequency was positively associated with more CUD symptoms and greater pain severity and not significantly associated with changes in insomnia, depression, or anxiety symptom severity.

Few studies assess the development of CUD in individuals using cannabis for medical purposes. The current study found that after 1 year of cannabis use, 11.7% of all participants and 17.1% of the daily or near-daily cannabis users had a CUD diagnosis, with 2 participants meeting criteria for severe CUD (6 symptoms). CUD at screening or baseline was exclusionary, so these were all new onset courses of CUD. Epidemiologic surveys of recreational cannabis use have indicated 3 in 10 adults who use cannabis develop CUD (36). Though prevalence in the current study is lower than the 30% 12-month incidence of CUD reported in Hasin et al. (36), it nonetheless indicates that individuals using cannabis for medical reasons may be at risk for CUD. Most current medical cannabis card regulations do not require a follow-up appointment with a certified physician after obtaining a medical cannabis card. This lack of follow-up differs from standard medical practice when prescribing other medications for these conditions such as antidepressants, opioids, and benzodiazepines. Due to the risk for CUD among individuals who use cannabis for medical concerns, a follow-up appointment with the prescribing physician may be warranted to assess balance between symptom improvement and emergence of CUD symptoms.

For those with a CUD diagnosis, the most frequently reported CUD symptoms were tolerance, using despite experiencing problems, spending a lot of time using, and craving. We recognize that there remains controversy in the field about whether a CUD diagnosis is appropriate for patients using cannabis for medical symptoms, rather than recreational purposes alone. For those taking *prescription* medications in the context of appropriate medical treatment, tolerance and withdrawal do not count as criteria for a substance use disorder. We note, however, that cannabis is not obtained *via* a prescription, but rather, through a recommendation. Thus, the system created for the regulation and distribution of cannabis for medical purposes is unique; unlike FDA-approved medications, the physician recommending cannabis has no authority over amounts, concentration, doses, or frequency of cannabis use for the patient and often no clinical guidance. Further, for many cannabis users, there is a blurred line between medical and recreational motives (e.g., in those using cannabis for “relaxation” purposes). Therefore, we did not discount tolerance or withdrawal as CUD symptoms in study participants.

The association between more frequent cannabis use and increased pain should be interpreted with caution, as it is unlikely that cannabis use caused or exacerbated pain. Instead, it is possible that individuals experiencing more pain used cannabis more frequently to treat their pain. The association between greater cannabis use and greater pain likely indicates that cannabis is not adequately treating pain symptoms. This viewpoint is supported by a recent position paper from the International Association for the Study of Pain (IASP) that found, after a comprehensive review of research on the use of cannabinoids to treat pain, there was a lack of sufficient evidence to endorse the general use of cannabinoids for the treatment of pain (50). Further, lack of improvement in symptoms of anxiety after 12 months of cannabis use adds to a growing body of literature that does not endorse cannabis as a treatment for these conditions (51). Though there was no significant worsening of symptoms, additional work suggests heavy cannabis use may increase risk for depression (52) and other psychiatric illnesses (53), particularly among adolescents and young adults (54–56). The lack of benefit from cannabis indicates that individuals with these chronic conditions should consider evidence-based treatments. Additionally, because there was no placebo cannabis, and because all participants were seeking cannabis as a potential therapeutic, the trial design created bias toward finding a treatment effect attributable to expectancy. This strengthens our confidence in the null findings for improvement in pain, anxiety, and depression symptoms as a function of frequency of cannabis use. We do note that depression symptoms improved in all participants over time; though there was no significant effect of cannabis frequency, future studies should include non-using control participants in order to tease apart the effects of any cannabis use from the effect of time or study procedures that involve reflecting on and discussing symptoms, which may itself lead to reduction in symptoms (57).

Although we hypothesized improvements in insomnia symptoms, increased frequency of cannabis use did not predict greater improvement in insomnia. There was a main effect of group on insomnia symptoms, driven by improvement in the RCT phase in the immediate card acquisition group (29), but no additional benefit over the 9-month post-randomized period. Interestingly, though participants in the immediate card acquisition group experienced a short-term benefit of cannabis for sleep, their sleep did not continually improve during the 9-month post-randomized period (Supplementary Figure 2). This is in line with prior work on sleep and cannabis use which suggests an initial benefit to insomnia but disruptions in sleep quality if cannabis is used long term (32, 33).

This study should be interpreted in light of its limitations. First, the sample was predominantly female and white which may limit the generalizability of our findings. We used a pragmatic design, meaning that participants chose which cannabis products and how much they used; therefore, this study cannot determine the effect of specific cannabinoids on symptoms of these disorders. It will be important for future studies to quantify which doses and constituents of cannabinoids may be therapeutic. Further, though most participants received a medical cannabis card from a doctor, few received adequate advice on product choice and dosing, largely because the evidence for specific products and doses is lacking. Therefore, it is possible participants were not using cannabis at therapeutic doses. Even so, current regulations state that after receiving a medical cannabis card, individuals may choose their products and dosing, lending ecological validity to this study. We did not assess quality-of-life measures such as stress levels, activity levels, or positive affect. Other studies suggest that even if symptoms themselves do not improve, cannabis may improve these quality-of-life measures (58, 59). Finally, past CUD (>1 year before enrollment) was not exclusionary for this study, though we note that rates of past CUD were low (8.0% of participants) and the time between any CUD diagnosis and trial enrollment was often long (M = 23, SD = 20 years prior to study enrollment).

In conclusion, in this 9-month follow-up study of a 12-week randomized clinical trial of medical cannabis card ownership, we found an association between more frequent cannabis use and increased CUD risk, with no significant improvement in pain, anxiety, insomnia, or depression symptom severity as a function of cannabis use. The current findings call into question the long-term utility of cannabis as an effective tool in relieving clinical symptoms.

## Data availability statement

The raw data supporting the conclusions of this article will be made available by the authors, without undue reservation.

## Ethics statement

The studies involving human participants were reviewed and approved by the Massachusetts General Brigham Institutional Review Board. The patients/participants provided their written informed consent to participate in this study.

## Author contributions

JG, RS, and AE contributed to the conceptualization and design of the original study. JG, MC, KP, BT-C, and BH designed the current research question and data analyses. JJ, MP, GP, and MC assisted with data collection. KP and MP organized and cleaned the data. KP performed the statistical analyses. MC, KP, and JJ wrote the first draft of the manuscript. JG and AE provided funding for data collection and salary support. All authors contributed to manuscript revision, read, and approved the submitted version.
