# Supplementary material for: Development of cannabis use disorder in medical cannabis users: A 9-month follow-up of a randomized clinical trial testing effects of medical cannabis card ownership
Source: Front Psychiatry. 2023 Mar 7;14:1083334. doi: 10.3389/fpsyt.2023.1083334 (PMC10027723; doi:10.3389/fpsyt.2023.1083334)

Supplemental Table 1: Cannabis use endorsement rates by group and visit.

| Cannabis use | Immediate | Delayed |
| --- | --- | --- |
| Prior to RCT trial (Month 0) | 96 | 67 |
| Less than monthly | 59.4% (57) | 47.8% (32) |
| Less than weekly | 19.8% (19) | 17.9% (12) |
| 1 - 4 days | 18.8% (18) | 32.8% (22) |
| 5 or more days | 2.1% (2) | 1.5% (1) |
| End of RCT trial (Month 3) |  |  |
| Less than monthly | 7.3% (7) | 46.3% (31) |
| Less than weekly | 7.3% (7) | 9% (6) |
| 1 - 4 days | 55.2% (53) | 34.3% (23) |
| 5 or more days | 30.2% (29) | 10.4% (7) |
| Use increased over prior 3 months | 79.2% (76) | 25.4% (17) |
| Use decreased over prior 3 months | 2.1% (2) | 14.9% (10) |
| Follow-up (Month 6) |  |  |
| Less than monthly | 13.5% (13) | 31.3% (21) |
| Less than weekly | 4.2% (4) | 17.9% (12) |
| 1 - 4 days | 53.1% (51) | 26.9% (18) |
| 5 or more days | 29.2% (28) | 23.9% (16) |
| Use increased over prior 3 months | 12.5% (12) | 34.3% (23) |
| Use decreased over prior 3 months | 21.9% (21) | 17.9% (12) |
| Follow-up (Month 12) |  |  |
| Less than monthly | 18.8% (18) | 26.9% (18) |
| Less than weekly | 12.5% (12) | 10.4% (7) |
| 1 - 4 days | 40.6% (39) | 29.9% (20) |
| 5 or more days | 24% (23) | 17.9% (12) |
| Use increased over prior 6 months | 10.4% (10) | 19.4% (13) |
| Use decreased over prior 6 months | 28.1% (27) | 19.4% (13) |

Supplemental Table 2: Main effects of time and randomization group and their interaction for each clinical outcome.

| Outcome | Effect | Cohen's D | Post. p-value |
| --- | --- | --- | --- |
|  |  | Mean; 95% CI |  |
| HADS [Anxiety] | Main effect of time | -0.11; -0.24 to 0.02 | p = 0.109 |
|  | Main effect of group | -0.07; -0.27 to 0.11 | p = 0.467 |
|  | Time x group interaction | 0.00; -0.01 to 0.02 | p = 0.528 |
| HADS [Depression] | **Main effect of time** | **-0.13; -0.26 to -0.01** | **p = 0.032** |
|  | Main effect of group | -0.10; -0.36 to 0.00 | p = 0.476 |
|  | Time x group interaction | 0.08; -0.07 to 0.35 | p = 0.562 |
| AIS | Main effect of time | -0.10; -0.25 to 0.04 | p = 0.182 |
|  | **Main effect of group** | **-0.30; -0.53 to -0.08** | **p = 0.008** |
|  | Time x group interaction | 0.01; 0.00 to 0.03 | p = 0.189 |
| BPI [Severity] | Main effect of time | -0.01; -0.09 to 0.08 | p = 0.891 |
|  | **Main effect of group** | **0.15; 0.03 to 0.27** | **p = 0.012** |
|  | Time x group interaction | 0.00; -0.07 to 0.05 | p = 0.891 |
| CUD symptoms | Main effect of time | 0.09; -0.36 to 0.54 | p = 0.695 |
|  | Main effect of group | 0.63; 0.00 to 1.31 | p = 0.185 |
|  | Time x group interaction | 0.05; -0.26 to 0.39 | p = 0.758 |

Supplemental Figure 1: Upset plot showing combinations of cannabis use disorder (CUD) symptoms for all participants and visits in which a CUD diagnosis was made. The bar plot on the bottom left panel provides marginal frequencies by symptom, the bar plot on the top panel reports frequencies for each combination of symptoms, and the line and dot plot on the bottom right shows the specific combination of symptoms per frequency.
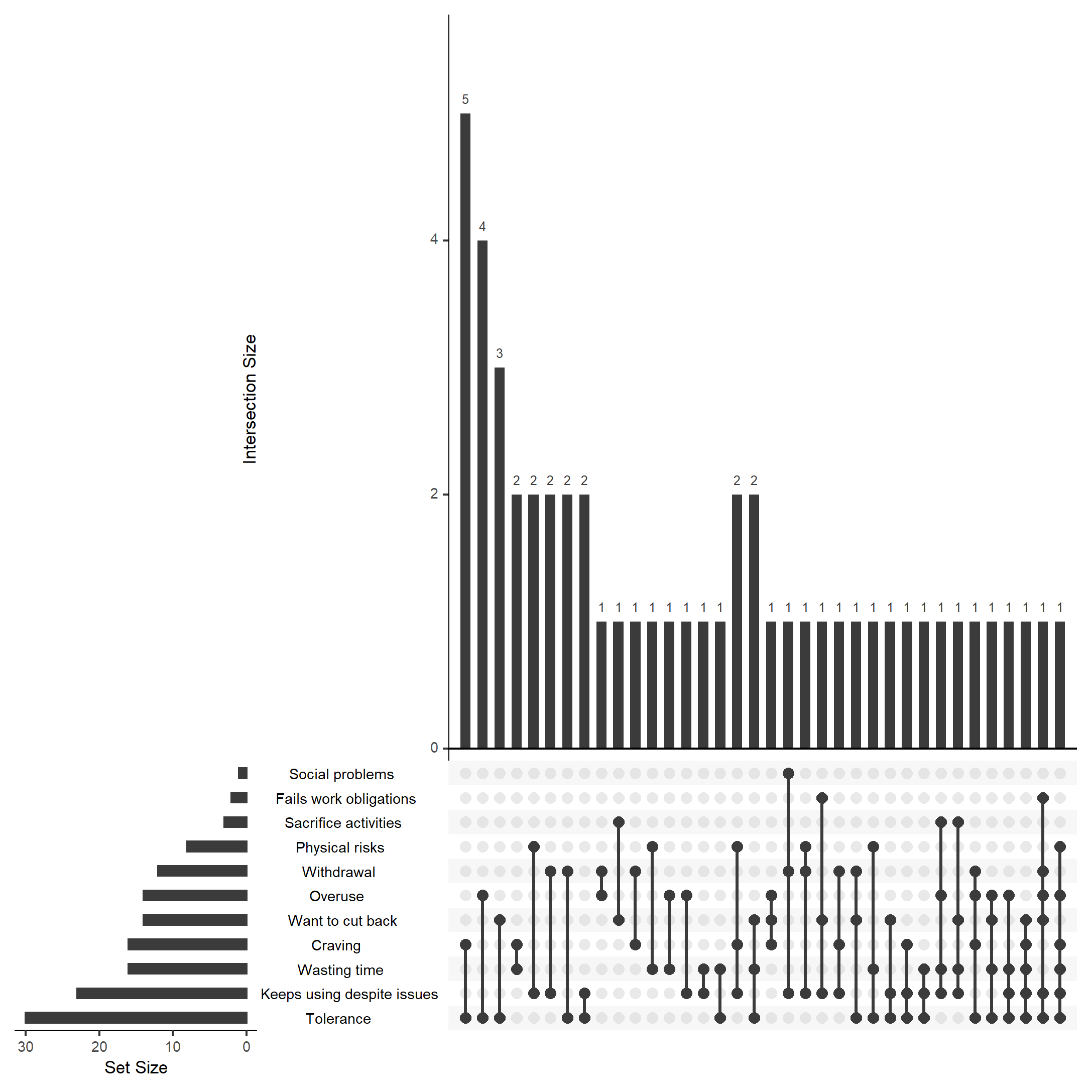


Supplemental Figure 2: Average AIS scores and 95% Cis for the immediate (orange) and delayed (blue) card acquisition groups across the follow-up period.


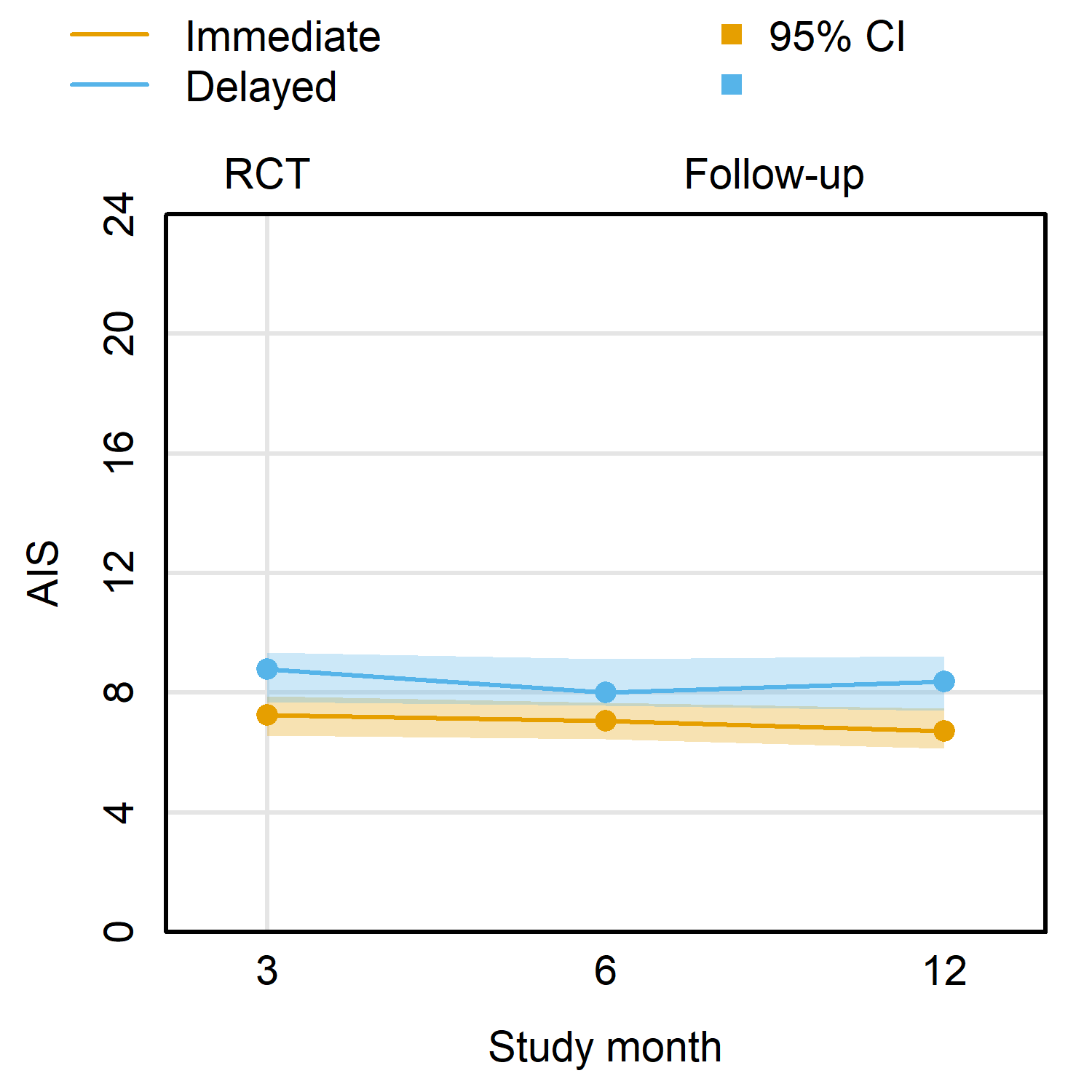

Supplement: Supplementary file 1 [file Data_Sheet_1.docx]
